# Knowledge about COVID-19 and Practices among Hemodialysis Technicians in the COVID-19 Pandemic Era

**DOI:** 10.1155/2020/6710503

**Published:** 2020-10-16

**Authors:** Amit S. Pasari, Amol Bhawane, Manish R. Balwani, Priyanka Tolani, Vishal Ramteke, Nishant Deshpande

**Affiliations:** ^1^Department of Nephrology, Jawaharlal Nehru Medical College, Sawangi, Wardha, Maharashtra, India; ^2^Department of Medicine, NKPSIMS, Nagpur, Maharashtra, India; ^3^Department of Nephrology, Kingsway Hospital, Nagpur, Maharashtra, India; ^4^Department of Nephrology, Alexis Hospital, Nagpur, Maharashtra, India

## Abstract

**Introduction:**

Hemodialysis technicians play a crucial role in infection control practices in hemodialysis units. Thus, it is important to assess the knowledge and attitude towards COVID-19 among hemodialysis technicians in this pandemic situation.

**Materials and Methods:**

An online survey composed of 22 closed-ended questions using Google Forms was conducted in the month of April (13th to 19th) 2020. The survey consisted of questions regarding the knowledge of COVID-19 and current hemodialysis practice among hemodialysis technicians. The study was approved by the institutional ethics board. The survey was administered online through a mobile phone invitation. Basic statistics (mean and standard deviation or total number and percent) were computed for all covariates.

**Results:**

Out of 150, 115 technicians participated in the survey. 80.9% of the participants were males. The mean age of respondents was 28.22 + 6.97 years. Most of the respondents could correctly identify fever (87.8%), breathlessness (86.08%), and dry cough (81.7%) as the symptoms of COVID-19 infection. 75.7% of the technicians were aware that it can be transmitted by asymptomatic persons. 61.1% of the technicians were segregating patients who had symptoms such as fever and cough to the last shift of the day. 81.1% of the technicians read the guidelines issued by the Indian Society of Nephrology—COVID-19 working group. But, only 25.5% of the respondents could rightly identify to keep a minimum distance of two meters between two beds while dialyzing a suspected patient of COVID-19 along with other patients to minimise risk of COVID-19 transmission. 60% of the technicians have received hydroxychloroquine as prophylaxis against coronavirus infection.

**Conclusion:**

Our study shows a significant knowledge gap among hemodialysis technicians about COVID-19. Effective COVID-19 education campaigns should be carried out intensively with relevant information among hemodialysis technicians to address the knowledge gap. A well-informed hemodialysis technician can prove to be a great tool to spread the right infection control practices among dialysis-dependent patients.

## 1. Introduction

Maintenance hemodialysis is a life-saving procedure for end stage renal disease (ESRD) patients. Most of these patients also suffer from comorbidities such as diabetes mellitus and hypertension. Such patients are at higher risk of severe COVID-19 infection [1]. Understanding of the transmission risk of COVID-19 is incomplete. The most common way of spread is thought to occur via respiratory droplets. Infection can also occur if a person touches an infected surface and then touches his or her eyes, nose, or mouth. The incubation period for COVID-19 is thought to be within 14 days following exposure [2].

In this situation, till no vaccine or definitive antiviral treatment is available, and social distancing is the norm. ESRD patients have to commute for dialysis two to three times per week and, thus, are at increased risk of exposure to the virus. It is equally important that hemodialysis technicians should be well aware of the disease pattern and methods to prevent its transmission. We tried to understand their current understanding of COVID-19 disease and hemodialysis practices through an online questionnaire.

## 2. Materials and Methods

An online survey composed of 22 closed-ended questions using Google Forms was conducted in the month of April (13th to 19th) 2020. The survey consisted of questions regarding the knowledge of COVID-19 and current hemodialysis practice in this pandemic situation. This study was approved by the institutional ethics committee at Jawaharlal Nehru Medical College, Sawangi, Wardha. Using Google Forms, we surveyed hemodialysis technicians practicing in Central India. The survey was administered online through a mobile phone invitation. Demographic and professional information of the hemodialysis technicians was obtained. Each participant was sent a unique link to the online survey. Participants were informed about the goal of the survey (medical research) in the preface of the questionnaire. By voluntarily participating in the survey after being given adequate information of its purpose, informed consent was implied. We confirm that participation was voluntary; participants could not be identified from the material presented and no plausible harm to participating individuals could arise from the study. Responses were made on a single Web page with one “submit” button that only allowed submissions through these unique links, thus making noninvited responses extremely unlikely. The survey questionnaire, before being administered, was validated properly. A pilot testing was performed on 10 technicians who did not participate in development of survey. The contents of the survey consisted of a questionnaire regarding the knowledge, attitudes, and practices towards COVID-19 in hemodialysis units. We sent the questionnaire to 150 hemodialysis technicians in Central India via a link on mobile phones. All the questions were closed ended with multiple choice answers. Basic statistics (mean and standard deviation or total number and percent) were computed for all covariates.

## 3. Results

Out of 150, 115 technicians participated in the survey. 80.9% of the participants were males. The mean age of the respondents was 28.22 + 6.97 years.

### 3.1. Awareness about Symptoms

Most of the respondents could correctly identify fever (87.8%), breathlessness (86.08%), and dry cough (81.7%) as the symptoms of COVID-19 infection, while a few could identify sore throat (60.80%), diarrhoea (33.04%), and myalgia (39.13%) as the symptoms of COVID-19 infection.

### 3.2. Mode of Transmission

Maximum respondents opined that the virus can spread through hand shaking (82%) while 67.8% and 46.08% were aware of the spread through droplets and fomites, respectively. 75.7% of the technicians were aware that it can be transmitted by asymptomatic persons.

### 3.3. Virus Survival

Around one-third respondents (37.60%) were aware that the COVID-19 virus can survive for more than one day on plastic surfaces.

### 3.4. Hand Hygiene

Around 96.4% of the technicians usually ask their patients to wash hands with soap and water for 20 seconds prior to coming into the dialysis unit.

### 3.5. Mask

48.7% of the technicians were using an N 95 mask in hemodialysis units. 26.5% of the technicians were using a surgical mask, while 17.7% were using a cloth mask. 97.3% of the technicians were asking patients to wear a face mask while coming for hemodialysis.

### 3.6. Segregation

61.1% of the technicians were segregating patients who had symptoms such as fever and cough to the last shift of the day.

### 3.7. History of Contact/Travel

92.9% of the technicians were asking about the history of travel to high-risk areas for COVID-19 infection, before taking patients for hemodialysis.

### 3.8. Willingness to Care for a COVID-19-Positive Patient

70.9% of the technicians were voluntarily willing to care/dialyse COVID-19-positive patients.

### 3.9. Current Difficulties

During the ongoing lockdown, 61.1% of the respondents had to face some difficulty in commuting to the hemodialysis unit. Most of them had difficulty in getting public transport, and repeated permissions were required from government authorities while commuting.

### 3.10. Knowledge about Recent Guidelines

81.1% of the technicians read the guidelines issued by the Indian Society of Nephrology—COVID-19 working group. But, only 25.5% of the respondents could rightly identify to keep a minimum distance of two meters between two beds while dialysing a suspected patient of COVID-19 along with other patients to minimize risk of COVID-19 transmission.

### 3.11. Prophylaxis

60% of the technicians have received hydroxychloroquine as prophylaxis against coronavirus infection. 58.6% of the respondents felt they may contract COVID-19 infection from the hemodialysis unit.

## 4. Discussion

Hemodialysis technicians and ESRD patients are frequently exposed to each other. Both remain at high risk of getting infected and, thus, transmitting COVID-19 among other dialysis patients and hospital staff. To prevent the transmission, both patients and technicians should be aware of the disease transmission pattern and the ways to prevent its spread. In this effort, we tried to assess the knowledge about COVID-19 virus among dialysis technicians so that, with this study, we can appropriately target the study content to be shared among dialysis technicians in further educational programmes. This study has its own limitations as it is based on the knowledge of technicians and may not necessarily reflect the exact pattern of practice being carried out at the dialysis centers.

Most of the respondents could correctly identify fever (87.8%), breathlessness (86.08%), and dry cough (81.7%) as the symptoms of COVID-19 infection. It is very important for a technician to know the COVID-19 disease clinical manifestations in detail, as a technician can observe signs and symptoms while doing hemodialysis of a patient. Such patients can be tested for COVID-19 infection if recognised early by the technician, and thus, it will be helpful in preventing the transmission of disease.

COVID-19 virus is transmitted between people through respiratory droplets and contact routes [2–7]. Transmission can also occur through fomites around the infected person [8].

Maximum respondents in our study opined that the virus can spread through hand shaking (82%), while 67.8% and 46.08% were aware of the spread through droplets and fomites, respectively. 75.7% of the technicians were aware that it can be transmitted by asymptomatic persons. In our study, there is less awareness among dialysis technicians about the modes of spread of coronavirus which has to be addressed on an urgent basis.

Around one-third (37.60%) of respondents in our study were aware that the COVID-19 virus can survive for more than one day on plastic surfaces. In a study published by Van Doremalen et al., it was found that the virus could survive on plastic and stainless steel up to 72 hours after application to these surfaces [9].

Around 96.4% of the technicians usually ask their patients to wash hands with soap and water for 20 seconds prior to coming into the dialysis unit. Hand hygiene is a very important step to break the chain of transmission. 97.3% of the technicians were asking patients to wear a face mask while coming for hemodialysis. Wearing a face mask is important in a way that it will minimize the habit of frequently touching the mouth and nose.

48.7% of the technicians were using an N 95 mask in hemodialysis units. 26.5% of the technicians were using a surgical mask, while 17.7% were using a cloth mask. In this pandemic situation, triple layer surgical masks and N 95 masks are not easily available due to shortage. It is important for dialysis technicians to take adequate precautions while doing hemodialysis of patients. Dialysis technicians received training regarding PPE and COVID-19 infection/preventive practices beforehand. 61.1% of the technicians were segregating patients having symptoms of cough and fever to the last shift of the day. This should be the standard practice in all hemodialysis setups where isolation rooms are not available to dialyse suspected COVID-19 patients. Stress should be laid on history taking specific to COVID-19 by dialysis technicians. In this study, 92.9% of the technicians were asking about the history of travel to high-risk areas for COVID-19 infection, before taking patients for hemodialysis.

In this pandemic, where healthcare workers are getting exposed to coronavirus and are, thus, being isolated/quarantined, there will be shortage of trained manpower. All hemodialysis units should have adequate staff who are willing to do dialysis of COVID-19-positive patients. In our study, 70.9% of the technicians were voluntarily willing to care/dialyse COVID-19-positive patients.

Most of the countries are using lockdown as a measure to break the chain of transmission and prepare themselves for this pandemic. Due to this lockdown, 61.1% of the respondents had to face some difficulty in commuting to the hemodialysis unit. Most of them had difficulty in getting public transport, and repeated permissions were required from government authorities while commuting.

In this ever-changing situation where there are new developments in the knowledge of COVID-19 daily, all healthcare workers have to remain updated so that maximum relief can be provided to patients. In this study, 81.1% of the technicians read the guidelines issued by the Indian Society of Nephrology—COVID-19 working group. But, only 25.5% of the respondents could rightly identify to keep a minimum distance of two meters between two beds while dialyzing a suspected patient of COVID-19 along with other patients to minimize risk of COVID-19 transmission [10].

60% of the technicians have received hydroxychloroquine as prophylaxis against coronavirus infection. Side effects of hydroxychloroquine were not enquired among participants. 58.6% of the respondents said that they may contract COVID-19 infection from the hemodialysis unit. In this situation, regular proper training and updation of knowledge about disease can allay the fear of technicians.

In the end, the authors are of opinion that, in this era of a pandemic, when there is shortage of manpower, hemodialysis technicians can prove to be an important link for transmitting correct information about COVID-19 to the hemodialysis patients along with measures/methods to prevent the spread of disease in the community. In order to achieve this, we should train our technicians about COVID-19 disease and the ways to prevent its spread.

## 5. Conclusions

Conducting regular educational meetings with dialysis technicians is vital for updating their knowledge. Our study shows a significant gap in knowledge and prevalent practices. With increasing prevalence of COVID-19 infection, need of informed and updated hemodialysis technicians will increase further. The authors suggest that hemodialysis technicians should undergo continuous medical education programmes related to COVID-19 at frequent intervals and there is a need of the central licensing authority to monitor their skills and knowledge.

## Data Availability

The data used to support the findings of this study are available from the corresponding author upon request.
